# The Effect of *ACACB cis*-Variants on Gene Expression and Metabolic Traits

**DOI:** 10.1371/journal.pone.0023860

**Published:** 2011-08-26

**Authors:** Lijun Ma, Ashis K. Mondal, Mariana Murea, Neeraj K. Sharma, Anke Tönjes, Kurt A. Langberg, Swapan K. Das, Paul W. Franks, Peter Kovacs, Peter A. Antinozzi, Michael Stumvoll, John S. Parks, Steven C. Elbein, Barry I. Freedman

**Affiliations:** 1 Wake Forest School of Medicine, Winston-Salem, North Carolina, United States of America; 2 Interdisciplinary Center for Clinical Research, Leipzig University, Leipzig, Germany; 3 Clinical Research Center, Malmo General Hospital, Lund University, Malmo, Sweden; Hospital Universitario 12 de Octubre, Spain

## Abstract

**Background:**

Acetyl Coenzyme A carboxylase β (ACACB) is the rate-limiting enzyme in fatty acid oxidation, and continuous fatty acid oxidation in *Acacb* knock-out mice increases insulin sensitivity. Systematic human studies have not been performed to evaluate whether *ACACB* variants regulate gene expression and insulin sensitivity in skeletal muscle and adipose tissues. We sought to determine whether *ACACB* transcribed variants were associated with *ACACB* gene expression and insulin sensitivity in non-diabetic African American (AA) and European American (EA) adults.

**Methods:**

*ACACB* transcribed single nucleotide polymorphisms (SNPs) were genotyped in 105 EAs and 46 AAs whose body mass index (BMI), lipid profiles and *ACACB* gene expression in subcutaneous adipose and skeletal muscle had been measured. Allelic expression imbalance (AEI) was assessed in lymphoblast cell lines from heterozygous subjects in an additional EA sample (n = 95). Selected SNPs were further examined for association with insulin sensitivity in a cohort of 417 EAs and 153 AAs.

**Results:**

*ACACB* transcribed SNP rs2075260 (A/G) was associated with adipose *ACACB* messenger RNA expression in EAs and AAs (p = 3.8×10^−5^, dominant model in meta-analysis, Stouffer method), with the (A) allele representing lower gene expression in adipose and higher insulin sensitivity in EAs (p = 0.04). In EAs, adipose *ACACB* expression was negatively associated with age and sex-adjusted BMI (r = −0.35, p = 0.0002).

**Conclusions:**

Common variants within the *ACACB* locus appear to regulate adipose gene expression in humans. Body fat (represented by BMI) may further regulate adipose *ACACB* gene expression in the EA population.

## Introduction

Acetyl-CoA carboxylase α and β (ACC1/ACACA and ACC2/ACACB) catalyze the synthesis of malonyl-CoA, the substrate for fatty acid synthesis and a regulator of fatty acid oxidation. Increased malonyl-CoA concentrations inhibit carnitine palmitoyaltransferase-1 (CPT1) activity, decreasing the rate of fatty acid entry into mitochondria and subsequent fatty acid oxidation [1]. ACACB is the key regulator of the fatty acid oxidation pathway [2] and *Acacb* knock-out mice are reportedly protected against obesity and diabetes induced by high fat/high carbohydrate diets [3]. Continuous fatty acid oxidation in adipocytes of *Acacb* knock-out mice is one factor contributing to their high insulin sensitivity [4]. Human gene expression studies suggest that *ACACB* is abundantly expressed in both oxidative and lipogenic tissues [5], [6].

When dietary intake exceeds the storage capacity of adipose tissue, excess lipid is delivered as ectopic fat to skeletal muscle, liver, pancreatic β-cells, and cardiac muscle. The resulting organ dysfunction, known as “lipotoxicity”, results in impaired insulin action and glucose homeostasis, and ultimately type 2 diabetes; however, the cellular mechanisms and intermediates are not fully understood [7]–[11]. *Acacb* knock-out mice [2], [3], [4] and rats treated with *Acaca* or *Acacb* anti-sense nucleotide inhibitors [12] suggested a potential therapeutic target for *ACACB* in insulin resistance, obesity, metabolic syndrome, and type 2 diabetes. Alternations in nutritional status may also regulate ACACA and *ACACB* expression [13], [14].

*ACACB* single nucleotide polymorphism (SNP) rs4766587 [15], [16] is associated with an increased risk of metabolic syndrome. Interestingly, a common SNP rs2268388 within *ACACB* is reproducibly associated with type 2 diabetes-related proteinuria and end-stage renal disease in non-African American (AA) populations [6], [17]. Animal studies [3] suggest that the lack of strong association between *ACACB* variants and obesity/diabetes may be masked by the cross-regulation of *ACACB* expression and hormonal/nutritional status in insulin-sensitive tissues [18], [19]. Gene-nutrient interactions may further influence the expression of functional variants in *ACACB* and their relationships with insulin sensitivity [16]. The *ACACB cis* regulatory SNPs rs2075259 and rs2075263 were significantly associated with *ACACB* messenger RNA levels in skeletal muscle (p = 3.0×10^−8^ and p = 5.2×10^−7^, respectively) [20].

We hypothesized that: (a) *cis* SNPs may regulate *ACACB* expression in insulin sensitive tissues and by consequence affect insulin sensitivity, and (b) body fat, represented by body mass index (BMI), may regulate *ACACB* expression in insulin responsive tissues. Therefore, we investigated the association between transcribed SNPs in *ACACB* and subcutaneous adipose tissue and skeletal muscle *ACACB* gene expression, as well as insulin sensitivity in European Americans (EAs) and AAs. We also tested for relationships between BMI and *ACACB* messenger RNA expression in adipose and skeletal muscle tissues.

## Results

Demographic characteristics of study subjects are listed in Table 1.

**Table 1:** Demographic and laboratory characteristics of study sample.

Sample	Race	n (M/F)	Age	BMI	TG	TC	HDL-C	S_I_
			(yr)	(kg/m^2^)	(mg/dl)	(mg/dl)	(mg/dl)	(×10^−4^ min^−1^[µU/ml]^−1^ )
Biopsy Sample	EA	105 (41/64)	40.3±11.2	28.9±5.91	132±123	183±35	53.7±18.3	3.70±2.04
	AA	46 (29/17)	43.5±9.25	30.0±6.33	109±65	175±40	51.4±12.7	3.29±1.85
Metabolic Sample	EA (all)	417 (163/254)	38.3±10.3	29.3±6.1	NA	NA	NA	5.85±5.97
	EA(AR)	293 (112/181)	37.6±9.85	29.8±6.0	NA	NA	NA	4.08±3.0
	EA(UT)	124 (51/73)	40.1±11.1	27.8±5.9	NA	NA	NA	9.70±8.75
								
	AA	153(68/85)	39.2±9.4	30.6±6.3	NA	NA	NA	3.70±3.55

Data expressed as mean±SD. EA, European American; AA, African American; AR, Arkansas; UT, Utah; M, male; F, female; NA, not available;

TG, triglyceride; TC, total cholesterol; HDL-C, HDL cholesterol; S_I_, insulin sensitivity.

### *ACACB* transcribed SNPs are associated with adipose *ACACB* messenger RNA expression

**Figure 1:**
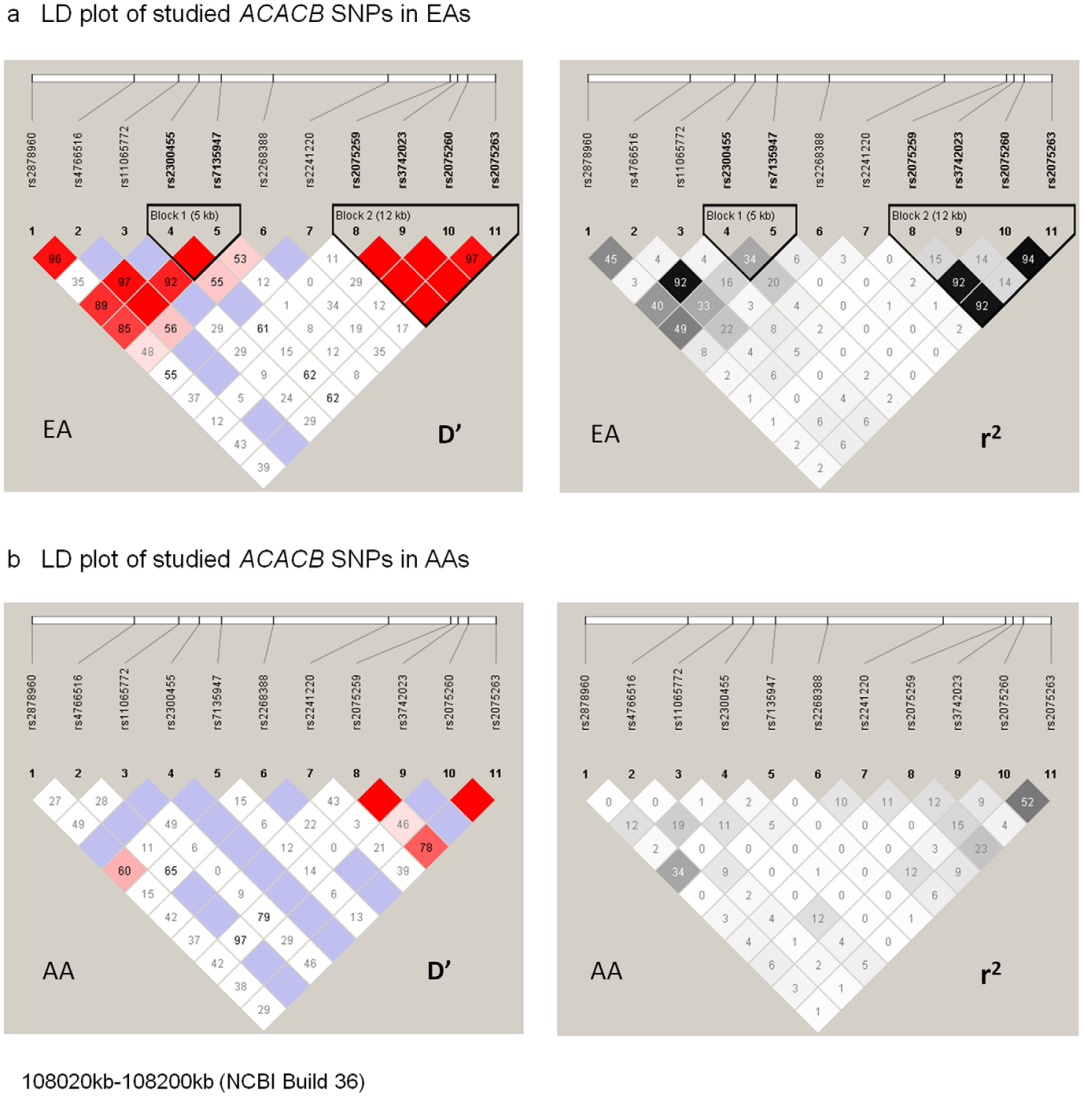
Linkage Disequilibrium (LD) plot of studied *ACACB* SNPs. 1a: Linkage Disequilibrium (LD) plot of studied *ACACB* SNPs in European Americans. 1b. Linkage Disequilibrium (LD) plot of studied *ACACB* SNPs in African Americans.

Eleven *ACACB* SNPs were genotyped in 105 EA and 46 AA subjects who were non-diabetic at the time of adipose and skeletal muscle biopsies. The linkage disequilibrium (LD) plots are shown in Figure 1. The LD pattern in EAs was similar to HapMap Caucasians, but differed from AAs and HapMap Yoruba Africans (Figure S1). The patterns of *ACACB* LD in AAs reflected those reported in HapMap for Yoruba Africans, with the exceptions of rs2075260 and rs2075263 for which the observed level was midway between HapMap Caucasians and Yoruba.

**Figure 2:**
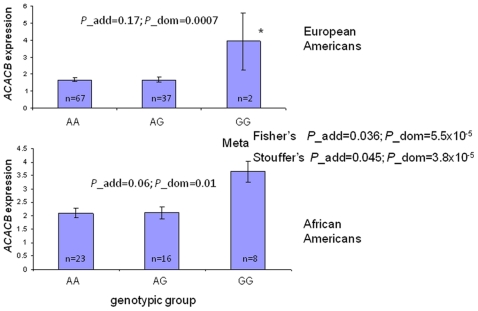
Adipose *ACACB* expression vs. rs2075260 in European Americans (EA) and African Americans (AA). Data are presented as least squares mean±SE after controlling age, sex, and BMI. * The ends of the error bar are the only two data points of *ACACB* expression values (adjusted for age, sex, and BMI) for this genotypic group. P values were adjusted for age, sex, and BMI. Fisher and Stouffer's methods were used for meta-analysis.

In subcutaneous adipose tissue, SNPs rs7135947, rs2075259, rs2075260, and rs2075263 were nominally associated with *ACACB* messenger RNA levels from the combined sample of EAs and AAs, after adjusting for age, sex, BMI, and race (p = 0.02-0.05, additive model; Table 2). The association of rs2075260 with *ACACB* expression was seen in EAs and AAs with the same direction of effect (p = 0.0007 and 0.01 respectively after adjusting for age, sex, and BMI; dominant model) (Table 2). When combining the association p-values in the two races by meta-analysis, the best p-value for association of rs2075260 with *ACACB* gene expression reached 5.5×10^−5^ (dominant model, Fisher's method) and 3.8×10^−5^ (dominant model, Stouffer's method) (Figure. 2). SNP rs7135947 was nominally associated with *ACACB* expression in AAs after adjusting for age, sex, and BMI (p = 0.04, additive model; Table 2). The association was not statistically significant in EAs, although the trend of expression versus genotype was consistent (Table 2). Since rs2075260 and rs7135947 are not in LD (r^2^≤1 in both EAs and AAs), when accounting for the additive effect of the eQTL-increasing alleles (G for rs2075260 and C for 7135947), the adjusted *ACACB* expression level was positively associated with the sum of eQTL-increasing alleles in AAs (p = 0.0046), as well as in EA and AA combined biopsy sample (p = 0.0001), but not in EAs alone (p = 0.11) (Figure S2; adjusted *ACACB* expression levels were controlled for age, sex, BMI, and ethnicity). Association between rs3742023 and *ACACB* expression was present in AAs, but not EAs (Table 2).

**Table 2:** *ACACB* messenger expression vs. genotype in adipose and muscle tissues in EAs and AAs.

No.	SNP ID	allele 1/2	Freq 1	11_exp	11_SE	11_N	12_exp	12_SE	12_N	22_exp	22_SE	22_N	p_add	p_dom	p_rec	Ethniciity	p_add_c	Tissue
1	rs2878960	C/T	0.34434	**1.81**	0.31	10	**1.73**	0.13	53	**1.68**	0.15	43	0.71	0.78	0.74	EA	0.33	adipose
1	rs2878960	C/T	0.680851	**2.34**	0.22	21	**2.14**	0.21	22	**1.62**	0.51	4	0.22	0.26	0.37	AA		adipose
2	rs4766516	A/G	0.212264	**1.65**	0.69	2	**1.76**	0.15	41	**1.69**	0.12	63	0.77	0.73	0.93	EA	0.9	adipose
2	rs4766516	A/G	0.053191				**1.78**	0.46	5	**2.23**	0.16	42	0.37	0.37	NA	AA		adipose
3	rs11065772	C/T	0.852381	**1.73**	0.11	77	**1.63**	0.19	25	**2.18**	0.57	3	0.94	0.42	0.83	EA	0.2	adipose
3	rs11065772	C/T	0.478261	**1.93**	0.29	12	**2.08**	0.23	20	**2.59**	0.27	14	0.10	0.09	0.30	AA		adipose
4	rs2300455	C/T	0.783019	**1.62**	0.12	62	**1.86**	0.15	42	**1.63**	0.68	2	0.30	0.93	0.25	EA	0.37	adipose
4	rs2300455	C/T	0.989362	**2.19**	0.15	46	**1.7**		1				0.64	NA	0.64	AA		adipose
5	rs7135947	C/T	0.45283	**1.9**	0.21	22	**1.7**	0.13	52	**1.63**	0.17	32	0.34	0.55	0.33	EA	**0.05**	adipose
5	rs7135947	C/T	0.691489	**2.48**	0.2	23	**1.95**	0.22	19	**1.72**	0.45	5	**0.04**	0.28	0.045	AA		adipose
6	rs2268388	C/T	0.834951	**1.76**	0.12	72	**1.6**	0.19	28	**1.83**	0.57	3	0.65	0.84	0.55	EA	0.1	adipose
6	rs2268388	C/T	0.818182	**2.43**	0.18	30	**1.75**	0.29	12	**1.61**	0.74	2	0.04	0.47	0.03	AA		adipose
7	rs2241220	C/T	0.84434	**1.83**	0.11	74	**1.47**	0.17	31	**1.07**		1	0.06	0.49	0.07	EA	0.16	adipose
7	rs2241220	C/T	0.695652	**2.17**	0.22	23	**2.29**	0.25	18	**2.08**	0.48	5	0.97	0.79	0.83	AA		adipose
8	rs2075259	A/G	0.203883	**3.91**	0.67	2	**1.7**	0.15	38	**1.65**	0.12	63	0.10	0.39	**0.0009**	EA	**0.03**	adipose
8	rs2075259	A/G	0.423913	**2.28**	0.31	10	**2.54**	0.22	19	**1.8**	0.23	17	0.13	**0.03**	0.78	AA		adipose
9	rs3742023	A/G	0.379808	**1.74**	0.26	14	**1.66**	0.14	51	**1.79**	0.16	39	0.71	0.57	0.95	EA	0.08	adipose
9	rs3742023	A/G	0.155556	**1.57**	0.66	2	**1.39**	0.29	10	**2.47**	0.16	33	**0.003**	**0.0007**	0.40	AA		adipose
10	rs2075260	A/G	0.806604	**1.67**	0.11	67	**1.67**	0.15	37	**3.93**	0.66	2	0.17	**0.0007**	0.57	EA	**0.02**	adipose
10	rs2075260	A/G	0.659574	**2.04**	0.2	23	**1.98**	0.25	16	**3.01**	0.35	8	0.06	**0.01**	0.34	AA		adipose
11	rs2075263	C/T	0.193396	**3.93**	0.66	2	**1.66**	0.15	37	**1.68**	0.11	67	0.22	0.67	**0.0007**	EA	**0.05**	adipose
11	rs2075263	C/T	0.212766	**3.11**	0.61	3	**2.34**	0.27	14	**2.02**	0.18	30	0.08	0.15	0.12	AA		adipose
1	rs2878960	C/T	0.34434	**0.74**	0.1	10	**0.88**	0.05	53	**0.82**	0.05	43	0.90	0.56	0.26	EA	0.6	muscle
1	rs2878960	C/T	0.680851	**0.97**	0.09	21	**0.86**	0.09	22	**0.73**	0.2	4	0.22	0.40	0.28	AA		muscle
2	rs4766516	A/G	0.212264	**0.83**	0.18	2	**0.82**	0.05	41	**0.85**	0.04	63	0.67	0.64	0.94	EA	0.84	muscle
2	rs4766516	A/G	0.053191				**0.88**	0.19	5	**0.9**	0.06	42	0.92	0.92	NA	AA		muscle
3	rs11065772	C/T	0.852381	**0.86**	0.04	77	**0.75**	0.07	25	**0.97**	0.21	3	0.42	0.53	0.25	EA	0.57	muscle
3	rs11065772	C/T	0.478261	**0.79**	0.12	12	**1.02**	0.09	20	**0.81**	0.11	14	0.97	0.35	0.30	AA		muscle
4	rs2300455	C/T	0.783019	**0.84**	0.04	62	**0.84**	0.05	42	**0.83**	0.18	2	0.91	0.94	0.91	EA	0.81	muscle
4	rs2300455	C/T	0.989362	**0.91**	0.06	46	**0.5**		1				0.32	NA	0.32	AA		muscle
5	rs7135947	C/T	0.45283	**0.82**	0.07	22	**0.84**	0.05	52	**0.86**	0.06	32	0.71	0.78	0.73	EA	0.87	muscle
5	rs7135947	C/T	0.691489	**0.96**	0.08	23	**0.84**	0.09	19	**0.83**	0.2	5	0.32	0.69	0.29	AA		muscle
6	rs2268388	C/T	0.834951	**0.86**	0.04	72	**0.81**	0.07	28	**0.92**	0.18	3	0.76	0.71	0.61	EA	0.59	muscle
6	rs2268388	C/T	0.818182	**0.95**	0.08	30	**0.85**	0.12	12	**0.69**	0.31	2	0.34	0.50	0.40	AA		muscle
7	rs2241220	C/T	0.84434	**0.87**	0.04	74	**0.76**	0.06	31	**0.7**		1	0.09	0.69	0.09	EA	0.55	muscle
7	rs2241220	C/T	0.695652	**0.84**	0.09	23	**0.93**	0.1	18	**1.07**	0.19	5	0.25	0.33	0.33	AA		muscle
8	rs2075259	A/G	0.203883	**0.83**	0.25	2	**0.82**	0.05	38	**0.85**	0.04	63	0.74	0.73	0.96	EA	0.60	muscle
8	rs2075259	A/G	0.423913	**0.82**	0.13	10	**0.93**	0.09	19	**0.92**	0.1	17	0.57	0.82	0.45	AA		muscle
9	rs3742023	A/G	0.379808	**0.91**	0.09	14	**0.81**	0.05	51	**0.86**	0.05	39	0.90	0.68	0.40	EA	0.70	muscle
9	rs3742023	A/G	0.155556	**0.67**	0.26	2	**1.07**	0.12	10	**0.84**	0.06	33	0.50	0.22	0.43	AA		muscle
10	rs2075260	A/G	0.806604	**0.86**	0.04	67	**0.81**	0.06	37	**0.84**	0.26	2	0.52	0.99	0.49	EA	0.42	muscle
10	rs2075260	A/G	0.659574	**0.83**	0.08	23	**0.81**	0.09	16	**1.27**	0.14	8	**0.03**	**0.003**	0.30	AA		muscle
11	rs2075263	C/T	0.193396	**0.84**	0.26	2	**0.82**	0.06	37	**0.85**	0.04	67	0.64	0.62	0.99	EA	0.51	muscle
11	rs2075263	C/T	0.212766	**1.4**	0.24	3	**0.92**	0.1	14	**0.84**	0.07	30	0.07	0.22	**0.03**	AA		muscle

EA, European American; AA, African American. P values were adjusted by age, sex, BMI.

p_add, p value under dominant model; p_dom, p value under dominant model; p_rec, p value under recessive model; p_add_c, p value of combining EA and AA by additional adjustment of race.

11_exp, 12_exp, 22_exp: *ACACB* relative expression levels in subjects with genotype 11, 12, and 22 respectively. Freq1: Frequency of allele 1; SE: standard error; N: number of subjects.

In skeletal muscle, rs2075260 and rs2075263 were nominally associated with *ACACB* messenger RNA levels in AAs, after adjusting for age, sex, and BMI (p = 0.03-0.003; additive and dominant/recessive models). However, association was not replicated in EAs and no evidence of association was observed in the combined biopsy sample for any of the tested SNPs (Table 2).

### Allelic expression imbalance of *ACACB* transcribed SNPs in lymphoblast cell lines

**Figure 3:**
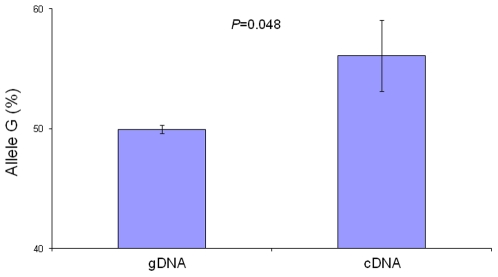
Allelic Expression of G of rs2075260 in both genomic and cDNAs of Utah EA lymphoblast cell lines (n = 18).

To identify a potential regulatory effect of *ACACB* transcribed SNPs, we applied an independent method to test AEI for six of the eight coding variants in lymphoblast cell lines from 95 Utah EAs under controlled cell culture conditions. In this group, 18 subjects were heterozygous for rs2075260 (A/G), where the mean (± SE) normalized percentage of G allele expression in cDNA was 56.08±2.97%, significantly higher than observed in the genomic DNA background (49.96±0.34%; *p* = 0.048; see Figure 3). Other SNPs were either not significant or not consistent with adipose/skeletal muscle gene expression (data not shown).

### Association of insulin sensitivity with *ACACB cis* transcribed SNPs in the metabolic sample

**Figure 4:**
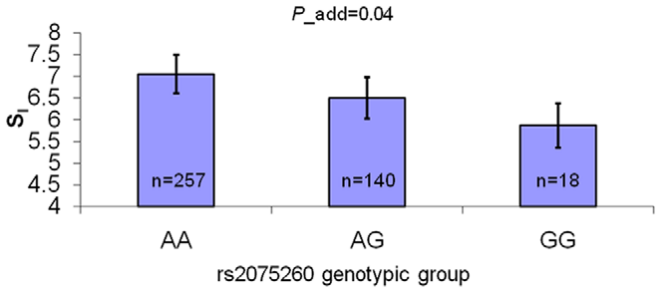
Insulin sensitivity by genotype of rs2075260 in European Americans. Data are presented as least squares mean±SE after controlling age, sex, BMI, cohort, and sibship. Additive *P*-value was adjusted for age, sex, BMI, cohort, and sibship (GEE model).

SNP rs2075260, which showed the strongest association between adipose *ACACB* expression for EAs and AAs in the biopsy sample, together with rs2075259 and rs2075263, which showed strong association with insulin sensitivity in 62 previously evaluated non-diabetic subjects (40 EA and 22 AA) (20), were further genotyped in the metabolic sample (416 EA and 153 AA). Rs7135947 was also genotyped in this sample due to significant association with adipose *ACACB* expression in AAs from the biopsy study sample. The association results with insulin sensitivity are shown in Table 3. Only rs2075260 was nominally associated with insulin sensitivity in EAs after adjusting for age, sex, BMI, sibship, and cohort (Utah or Arkansas) (p = 0.04, additive model). Allele G, associated with higher *ACACB* expression in the adipose biopsy sample (Figure 2) and AEI (Figure 3), was also associated with lower insulin sensitivity in EAs after appropriate adjustment (Figure 4). No significant association of this SNP was seen in AAs (additive p = 0.71; Table 3).

**Table 3:** Association between selected transcribed SNPs and insulin sensitivity (SI) in the metabolic sample.

SNP	Allele (1/2)	Race		S_I_ vs.Genotype/n		genetic power*	P_add	P_dom	P_rec	P_add_comb
			CC	CT	TT					
		EA	6.37±0.56	6.79±0.37	7.16±0.79		0.66	0.72	0.76	
rs7135947	C/T	n = 416	81	217	118	0.99				0.71
		AA	3.28±0.27	4.58±0.48	2.55±0.36		0.92	0.10	0.36	
		n = 153	73	64	16	0.60				
			AA	AG	GG					
		EA	6.39±0.72	6.69±0.46	6.94±0.45		0.83	1.00	0.33	
rs2075259	A/G	n = 411	17	148	246	0.99				0.75
		AA	3.23±0.42	3.65±0.32	3.93±0.44		0.60	0.74	0.52	
		n = 149	14	67	71	0.58				
			AA	AG	GG					
		EA	7.05±0.44	6.50±0.47	5.87±0.51		**0.04**	0.87	**0.046**	
rs2075260	A/G	n = 415	257	140	18	0.99				0.07
		AA	3.81±0.38	3.71±0.36	3.48±0.38		0.71	0.25	0.98	
		n = 152	79	61	12	0.59				
			CC	CT	TT					
		EA	5.62±0.66	6.59±0.47	6.99±0.43		0.58	0.57	0.94	
rs2075263	C/T	n = 416	12	134	270	0.99				0.68
		AA	3.90±0.58	3.75±0.36	3.73±0.36		0.41	0.61	0.20	
		n = 152	7	59	86	0.59				

EA European American; AA African American.

Data are presented as least squares mean±SE, after controlling for age, sex, and BMI.

Location (Arkansas/Utah) is additionally adjusted for in EA.

P_add reflects p-value for the additive model; P_dom reflects p-value for the dominant model; P_rec reflects p-value for recessive model.

P_add_comb is additionally adjusted for race when EA and AA are combined for analysis.

*Genetic power was estimated to detect 10% of the variation in insulin sensitivity and other metabolic traits in the metabolic sample(assuming a type 1 error rate = 0.0005).

### Adipose *ACACB* gene expression correlates with BMI in EA

**Figure 5:**
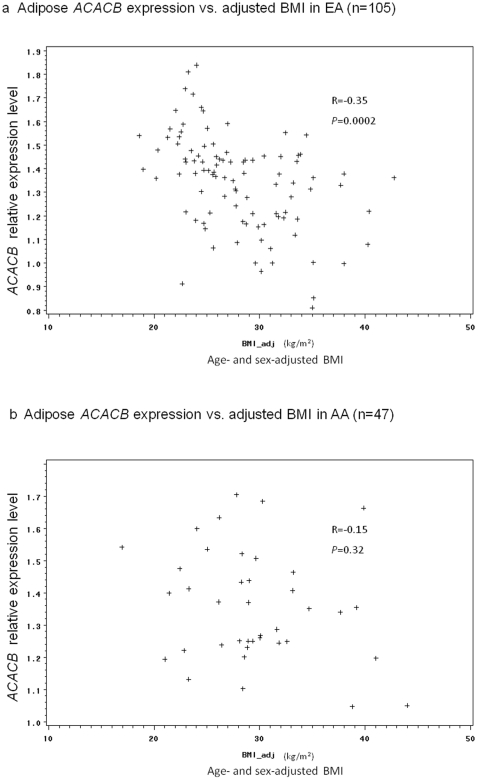
Adipose *ACACB* expression vs. adjusted BMI. Figure 5a Adipose *ACACB* expression vs. adjusted BMI in EA. Figure 5b Adipose *ACACB* expression vs. adjusted BMI in AA.

Adipose *ACACB* messenger RNA expression level was negatively associated with age and sex-adjusted BMI in EAs (r = −0.35, p = 0.0002; Figure 5a), but not in AAs (Figure 5b). Significant differences in BMI or S_I_ were not found between EAs and AAs in the biopsy sample (Table 4). However, adipose *ACACB* expression was significantly lower in EAs than AAs in the biopsy sample (Table 4).

**Table 4:** Comparison of *ACACB* expression, Si, and BMI between EAs and AAs in the biopsy. Sample.

	EA	AA	P value	Adjustment
	(n = 105)	(n = 46)		
Adipose ACACB expression	1.68±0.10	2.33±0.15	**0.0004**	age, sex, BMI
				
Adipose ACACB expression	1.76±0.10	2.37±0.17	**0.002**	age, sex, SI
				
SM ACACB expression	0.83±0.03	0.93±0.06	0.15	age, sex
				
SM ACACB expression	0.83±0.03	0.93±0.06	0.15	age, sex, BMI
				
SM ACACB expression	0.80±0.04	0.90±0.06	0.18	age, sex, SI
				
S_I_ (×10^−4^ min^−1^[µU/ml]^−1^ )	3.65±0.19	3.60±0.30	0.88	age, gender, BMI
				
BMI (kg/m^2^)	28.1±0.54	29.6±0.86	0.15	age, gender

Data are least squares mean±SE. The controlled covariates are listed in the adjustment column.

SM: skeletal muscle; EA: European American; AA: African American.

### Association of BMI, triglycerides (TG), total cholesterol (TC), and HDL cholesterol (HDL-C) with *ACACB cis* transcribed SNPs in the metabolic sample

No significant associations were identified with BMI, TG, TC or HDL-C for the selected SNPs in the metabolic sample. SNP rs2075260 showed the lowest p-values for association with BMI in EAs and AAs (dominant p = 0.19 and 0.10, respectively; Table S1); however, significant association was not observed in the combined EA and AA sample. No association was identified with TG, TC, and HDL-C (data not shown).

## Discussion

*ACACB* is a key rate limiting enzyme in mitochondrial fatty acid oxidation. In this study, we found that the *ACACB* transcribed SNP rs2075260 was associated with adipose tissue *ACACB* gene expression in EAs and AAs and with insulin sensitivity in EAs; the allele associated with lower gene expression was also associated with higher insulin sensitivity. This is consistent with the mouse model, which demonstrated that inhibition of *Acacb* improved insulin sensitivity [3]. These data suggest that *ACACB cis*-acting SNPs may regulate gene expression in humans, potentially altering insulin sensitivity independent of BMI. Although the association of rs2075260 with insulin sensitivity was not statistically significant in the AA sample, the effect of this allele was similar in magnitude and in the same direction as that observed in EAs. This result is similar to that seen in an independent German Sorb population sample whose LD pattern was similar to HapMap CEU with the association between rs2075263 (C/T) and homeostasis model assessment of insulin resistance (HOMA-IR; p = 0.22, beta = 0.06, n = 793) (personal communication: Stumvoll M, 2011) [21], as well as in Diabetes Genetics Initiative (DGI) for HOMA-IR (p = 0.03, beta = 0.11, n = 1393), where the meta-p value of these two cohorts reached 0.01 and 0.02 for Stouffer and Fisher's methods respectively with minor allele C indicating lower insulin sensitivity, consistent with rs2075260 in Table 3 where minor allele G represented lower insulin sensitivity in the AR and UT Caucasian metabolic sample . Our inability to detect association at this locus in AAs may reflect low statistical power in this small sample. Concerns about multiple testing are relevant in genetic studies. The number of SNPs to account for in this study is impacted by the fact that rs2075259, rs2075260, and rs2075263 are in high genotypic concordance in Caucasians. Given this, we presented the raw p values without adjustment for multiple comparisons.

The non-synonymous SNP rs2075260 (G/A) encodes an amino acid substitution Val2141Ile [NM_001093]. We are unable to predict ACACB protein structural changes caused by this amino acid substitution (http://snpeffect.vib.be/). We scanned this SNP using MatInspector (http://www.genomatix.de ) and TFSEARCH (http://www.cbrc.jp/ research/db/TFSEARCH.html) and have not identified potential anchoring loci for a transcription factor binding site at rs2075260. Therefore, we are unable to determine whether this SNP is a functional variant. However, rs2075259 (G/A), in high genotypic concordance with rs2075260, forms activating protein (AP1)/ v-maf musculoaponeurotic fibrosarcoma oncogene homolog (MAF) anchoring sites for allele G (matrix similarity = 0.96-0.98), while allele A abolished these potential binding sites. As we demonstrated, the G allele of rs2075259 is associated with lower *ACACB* gene expression in adipose tissue (Table 2). AP1/MAF may act as a suppressor, or impact other repressors, of gene expression [22], [23]. This is supported by the strong negative correlation between messenger level of *ACACB* and *MAF/AP1S2* (AP1 subunit 2) in our adipose gene global expression study in non-diabetic subjects (unpublished data).

No SNP was associated with BMI in the metabolic sample (Table S1). However, adipose *ACACB* expression was negatively associated with age and sex-adjusted BMI in EAs (Figure 5a). This correlation cannot be explained by the known regulatory function of *ACACB* on body weight and insulin sensitivity in the mouse model [2], [3], [4]. We suspect that BMI may regulate adipose *ACACB* expression in EAs. Although this cross-sectional study cannot provide direct evidence, this hypothesis is supported by our observation that HepG2 cell *ACACB* expression was down-regulated after treatment with 1 mM palmitate [13]. It is conceivable that nutritional stress (e.g. diet-indued obesity and free fatty acid exposure) regulates *ACACB* expression. Down regulation of *Acacb* was observed in visceral fat tissue in rats fed a high-fat diet, while *Juniperus chinensis* extract significantly reduced this effect (14). AMPK (AMP activated protein kinase) may be the link between *ACACB* gene expression and nutritional status [24]. ACACB activity [25] can be directly inhibited by AMPK. This may constitute a physiologic link between higher BMI and lower *ACACB* gene expression and supports the metabolic adaptation model proposed by Weyer et al based on energy expenditure, fat oxidation, and body weight regulation in Pima Indians [26]. The lack of association between adipose *ACACB* gene expression and BMI in AAs may be due to the smaller sample size or imprecision of BMI as a measure of adiposity. Alternatively, AAs may be less sensitive to nutritional stress in terms of regulating adipose *ACACB* expression. Adipose *ACACB* expression levels are significantly higher in AAs than EAs (Table 4). However, BMI and insulin sensitivity were nearly equivalent in EAs and AAs in the biopsy study sample (Table 4). This finding may reflect racial differences in *ACACB* expression in response to similar body fat. Different genetic or environmental factors may affect *ACACB* expression between EAs and AAs.

*ACACB* variants have not been shown to be associated with BMI or diabetes using GWAS. However, functional variants (e.g. transcribed SNPs) may still regulate gene expression and impact insulin sensitivity. Our biopsy sample was modest and generated approximately 86% power in EAs and 30% power in AAs to detect 20% of the variation in gene expression levels (assuming a type 1 error rate = 0.0005). Larger populations will be necessary to replicate these results. The lack of association of *ACACB* variants with BMI/diabetes may also reflect the masking effect of gene*gene and gene*environment interactions. A recent gene*environment interaction study of *ACACB* variants and metabolism suggested that uncovered causative variants may have been overlooked in published GWAS [16]. *Acacb* knock-out mice were only protected from obesity and diabetes when fed high fat/high-carbohydrate diets; when fed normal chow, significant differences in body weight were not observed [3].

Two independent groups reported that *Acacb* knock-out mice have similar body weights as wild type mice [27], [28]. Molecular explanations for the phenotypic differences observed between Olson's model of *Acacb* deletion and the original model (prone to resistant to obesity, diabetes and insulin resistance) published by Abu-Elheiga et al. [2] are not clear. Although the *Acacb* biotin-binding site was deleted in both models, different targeting strategies were employed. The targeting strategy employed in the original study replaced only the exon containing the biotin binding motif [2]. RNA splicing across the targeting cassette might leave the mRNA in frame, resulting in a mutated but otherwise intact protein lacking a catalytic domain. If produced, such a protein might potentially have “dominant negative” activity toward ACACA [27]. This may be supported by the effects of soraphen, an inhibitor of both ACACA and ACACB, improving peripheral insulin sensitivity in mice fed high-fat diets [29]. It suggests that lower *ACACB* expression alone may be insufficient to improve insulin sensitivity. As in the present study, association between rs2075260 and S_I_ was weaker compared to the association with gene expression. It is noteworthy that this result was based on transcript messenger level only, as protein level and enzyme activity data are not available yet. Limitations of this study included the small sample of AA subjects, lack of longitudinal follow-up data and lack of dietary intervention. These factors limit interpretation of gene expression effects. We also lack quantifiable life-style data to use as covariates in our database. Free fatty acid (FFA) levels fluctuate even with fasting. Healthy non-diabetic, non-drug or tobacco using subjects should be recruited on balanced diets for three days, with blood samples drawn after an overnight fast. We are not confident that our blood samples meet these criteria. Therefore, we elected not to measure FFAs in our sample. We were unable to test the effects of specific ACACB inhibitors on human insulin sensitivity. Finally, due to the large size of the *ACACB* gene (Ensembl transcript ID ENST00000338432: 55 exons spanning 150 kb), where 142 SNPs were available in HapMap database (NCBI builder 36), we prioritized the effect of transcribed SNPs on gene expression. The most significant SNP for adipose *ACACB* expression in the biopsy sample (rs2075260) was in high genotypic concordance with rs2075259 and rs2075263, which rank among the top SNPs in skeletal muscle eQTL mapping [20]. However, rs2075260 was not associated with skeletal muscle *ACACB* expression in the biopsy sample. This observation is not uncommon in terms of eQTLs in different cell types [30]. This could also result from different transcript forms presented across different cell types or variation in participant characteristics in the two study samples. In addition, the *ACACB* probe (Agilent expression array) applied in the eQTL mapping [20] was located at the 3′ UTR of the *ACACB* gene, while the expression primer set (for RT-PCR) used in the current study was designed across exon 13 and 14, in an attempt to cover all of the reported transcripts. Transcribed SNPs are a logical source for testing AEI as a complementary prioritization method for gene expression based on genotype. We chose lymphoblast cell lines for AEI studies instead of adipose tissue due to mixed sources of adipose RNA, as well as the restricted amount of tissue RNA available. It can be argued that lymphoblast cell lines are not the most relevant cell types to evaluate insulin action. However, our prior study revealed that lymphoblast cell lines provided confirmatory evidence for eQTLs in insulin-response tissues [31].

Our data suggest that the common transcribed SNP rs2075260 (A/G) in the coding region of the *ACACB* gene is associated with adipose tissue *ACACB* messenger RNA expression in EAs and AAs. The G allele, representing higher levels of gene expression, is also associated with lower insulin sensitivity in EAs. Body fat, represented by BMI, may serve as a “negative feed-back” signal down-regulating *ACACB* expression in adipose tissue, possibly as a mechanism of metabolic adaptation. Longitudinal intervention studies will assist in interpreting whether high *ACACB* expression is a risk factor for obesity and type 2 diabetes. Gene*gene interaction studies in larger cohorts will be helpful to identify any undetected diabetes or obesity genes that interact with *ACACB*. Analysis of alternative splicing of *ACACB* in adipose tissue and skeletal muscle may be helpful to address tissue-specific transcripts and regulatory SNPs involved in gene expression.

## Materials and Methods

### Subjects and phenotypes

The “biopsy sample” consisted of 105 EAs and 46 AAs from Little Rock, Arkansas who lacked diabetes based on ADA diagnostic criteria 2010 [32]. All participants underwent a screening visit during which height, weight, fasting blood lipids, and blood insulin and glucose concentrations (fasting, 30, 60 and 120 minutes after a standard 75-g oral glucose load) were measured, and adipose and skeletal muscle biopsies were performed using a Bergstrom needle under local (lidocaine) anesthesia at University of Arkansas for Medical Sciences (UAMS), Little Rock, Arkansas. Biopsy samples were immediately rinsed in normal saline, cut, and snap frozen in liquid nitrogen. Premenopausal women were studied in the follicular phase of the menstrual cycle.

The “metabolic sample” consisted of 440 non-diabetic EAs (417 with measures of insulin sensitivity) and 163 non-diabetic AAs (153 with measures of insulin sensitivity). AAs were recruited in Arkansas, whereas 293 EAs were recruited from Arkansas and 124 were siblings from 62 nuclear families of Northern European descent ascertained in Utah. Subjects in the previously described “biopsy sample” were a subset of the Arkansas “metabolic sample”. An insulin-modified (0.04 U/kg), frequently sampled intravenous glucose tolerance test (FSIGT) was performed, as reported [33]. Insulin sensitivity (S_I_) was calculated from the FSIGT using either the MinMod (Utah sample) or MinMod Millenium (Arkansas Sample) programs [34], [35]. These programs use the same algorithms and provide nearly identical estimates of S_I_.

Subjects provided written, informed consent under protocols approved by either the Institutional Review Board of the University of Utah Health Sciences Center or UAMS. Information from these subjects was de-identified and samples transferred to the Wake Forest University School of Medicine (WFUSM). This study was approved by the WFUSM Institutional Review Board.

### Laboratory measurements

Insulin levels were measured using an immunochemiluminometric assay (Molecular Light Technology, Wales, UK) and plasma glucose by a glucose oxidase assay. Standard clinical assays (lipids, glucose) were performed at LabCorp (Burlington, NC).

### Genotyping

Eight common transcribed *ACACB* SNPs were genotyped in the biopsy sample (rs2878960, rs4766516, rs11065772, rs2300455, rs7135947, rs2241220, rs3742023, rs2075260), all with minor allele frequencies >0.05 in Caucasians and Yoruba Africans based on HapMap data (http://hapmap.ncbi.nlm.nih.gov/). Additionally, rs2268388 (significantly associated with diabetic nephropathy) [6], [17] and rs2075259/rs2075263 (two top SNPs for *ACACB* expression quantitative trait loci [eQTL] in skeletal muscle) [20] were genotyped. Genotyping of the biopsy sample was performed on a PSQ 96 Pyrosequencer (Biotage, Uppsala, Sweden). Additional genotyping in metabolic sample participants was performed by pyrosequencing or the ABI TaqMan assay (Applied Biosystems, Foster City, CA). Genotype distributions for all variants met Hardy-Weinberg expectations (p>0.01). The overall genotype call rate was above 98%. Sixty-nine duplicated QC samples were randomly distributed across genotyping plates of the metabolic sample to assure 100% reproducibility. For the biopsy sample, in addition to the fact that pyrosequencing is a highly reliable genotyping method which visualizes and quantifies both alleles of target SNPs, AEI requires genotyping of both genomic and cDNAs, which virtually serve as duplicates to assure accuracy.

Linkage disequilibrium plots (D′ and r^2^) were generated using the HaploView program (http://www.broadinstitute.org/haploview).

### Gene expression

Total RNA was isolated from subcutaneous adipose tissue using the RNAeasy Lipid Tissue Mini Kit (QIAGEN, Valencia, CA) and from skeletal muscle using the Ultraspec RNA kit (Biotecx Laboratories, Houston, TX). The quantity and quality of isolated RNA were determined by ultraviolet spectrophotometry and electrophoresis, respectively, using the Agilent 2100 Bioanalyzer (Agilent Technologies, Santa Clara, CA), and 1 µg was reverse transcribed using random hexamer primers with Qiagen reverse transcribed reagents (QIAGEN, Valencia, CA). All RNA samples from a single study population and tissue were reverse transcribed using the same kit on the same day. The standard curves were generated using pooled RNA from assayed samples. Primers were designed to capture most known splice variants where the amplicon spanned an intron. *ACACB* expression was measured by real time PCR (SybrGreen) on an ABI 7500-Fast Real time-PCR system (Applied Biosystems, Foster City, CA) using 18S ribosomal RNA as a normalization standard. Primer sequences were as follows: 18S forward: ATCAACTTTCGATGG TAGTCG, 18S reverse: TCCTTGGATGTGGTAGCCG, ACACB– Forward: GGGCTCCTGCTCTCCTACA, ACACB – Reverse: CGTTCTCCTTCTCAAACACACA [36].

### Allelic expression imbalance (AEI)

Transformed lymphocytes were cultured from an independent sample of 95 unrelated HapMap Utah Caucasians (EAs) as reported [30]. Total RNA was extracted using the RNEasy mini kit (QIAGEN, Valancia, CA), quantity and quality was assessed on an Agilent 2100 Bioanalyzer (Agilent Technologies, Inc, Santa Clara, CA). Unequal expression of *ACACB* alleles was sought as evidence for *cis* acting regulatory variants by comparing peak heights in individuals heterozygous for the synonymous coding SNPs rs2878960, rs4766516, rs2300455, rs7135947, rs2241220, and rs2075260 [37]. Rs11065772 and rs3742023 failed in assay design. Briefly, total RNA was reverse transcribed using random hexamers. Allelic specific quantitation of both cDNA and genomic DNA samples was determined using the same assay for pyrosequencing on a PSQ 96 Pyrosequencer (Biotage, Uppsala, Sweden) with peak height quantified using Allele Quantification software (Biotage, Uppsala, Sweden).

### Statistical analysis

S_I_ was estimated from the insulin and glucose data using either the MinMod (Utah sample) or MinMod Millenium (Arkansas sample) programs. Gene expression levels were normalized to 18S RNA and the ratio was used in\all calculations. Statistical analyses were performed using the SAS 9.1 software of the SAS Institute (Cary, NC). Correlations between *ACACB* expression and BMI or S_I_ were assessed using a general linear model after controlling potential confounders (age, race, sex, BMI). To approximate normality, the logarithm of *ACACB* expression was used in all analyses. Allelic specific expression was assessed by comparing the percentage of normalized genomic and cDNA expression on observed alleles using the method of Fogarty et al [38]. For the biopsy samples, a generalized linear model (GENMOD) was used to assess the association between genotype and *ACACB* gene expression after adjusting for age, sex, and BMI, although age and sex were not obvious confounders (Table S2). For analysis under an additive model, homozygotes for the allele (1/1), heterozygotes (1/2), and homozygotes for the allele (2/2) were coded to a continuous variable (0, 1, and 2). The dominant model was defined as contrasting genotypic groups 1/1+1/2 vs. 2/2. The recessive model was defined as contrasting genotypic groups 1/1 vs. 1/2+2/2. The generalized estimating equations (GEE) procedure was used to account for sibships in the metabolic sample in addition to age, sex, BMI, and cohort, since Utah subjects were family-based. We analyzed each SNP using the same genetic model for all analyses, irrespective of the study sample. A meta-analysis was performed using Stouffer [39] and Fisher's method [40]. P-values <0.05 were considered to represent a nominal level of statistical significance.

## Supporting Information

Figure S1 **Linkage Disequilibrium (LD) plot of studied** ***ACACB*** **SNPs in HapMap.** S1a: Linkage Disequilibrium (LD) plot of studied *ACACB* SNPs in HapMap Caucasians. S1b. Linkage Disequilibrium (LD) plot of studied *ACACB* SNPs Yoruba Africans.
(TIF)

Figure S2 **Adjusted** ***ACACB*** **expression by sum of eQTL-increasing alleles in adipose.** Adjusted *ACACB* expression levels were controlled for age, sex, BMI, and ethnicity. eQTL-increasing alleles: G for rs2075260; C for 7135947. *P* values were obtained under general linear model (GLM). EA: European American; AA: African American; All: EA and AA combined.
(TIF)

Table S1 Association between selected transcribed SNPs and BMI in the metabolic sample. Data are least squares mean±SE, after controlling age and sex. Cohort location (Arkansas/Utah) is additionally adjusted for in EA.
(XLS)

Table S2 Summary of Type III testing for age and sex as individual coefficients of *ACACB* expression in the biopsy sample. EA: European American; AA: African American; DF: degree of freedom.
(XLS)
