# Supplementary figures and images for: The Effect of ACACB cis-Variants on Gene Expression and Metabolic Traits
Source: PLoS One. 2011 Aug 26;6(8):e23860. doi: 10.1371/journal.pone.0023860 (PMC3162605; doi:10.1371/journal.pone.0023860)

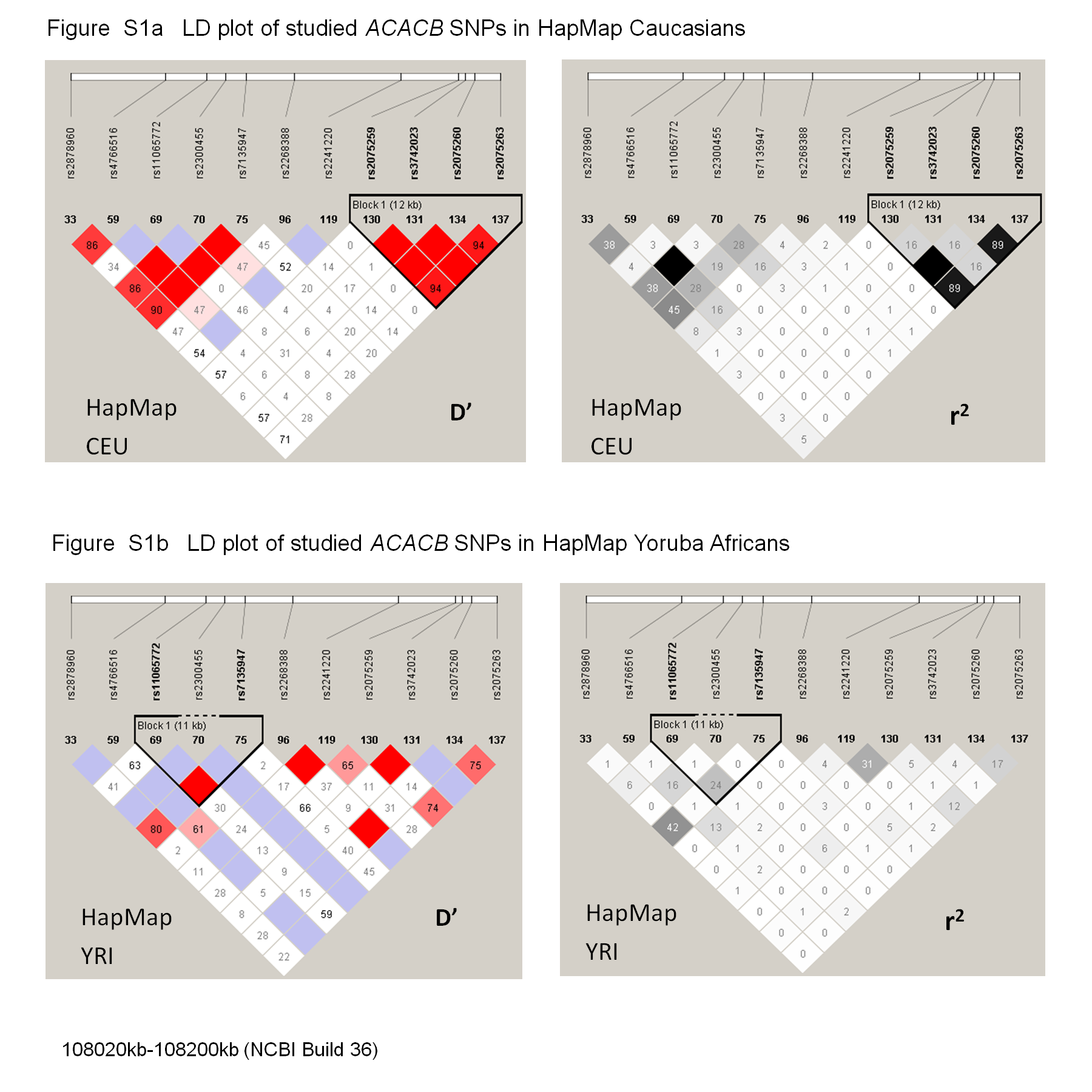

Supplement: Figure S1 — Linkage Disequilibrium (LD) plot of studied ACACB SNPs in HapMap. S1a: Linkage Disequilibrium (LD) plot of studied ACACB SNPs in HapMap Caucasians. S1b. Linkage Disequilibrium (LD) plot of studied ACACB SNPs Yoruba Africans. (TIF) [file pone.0023860.s001.tif]

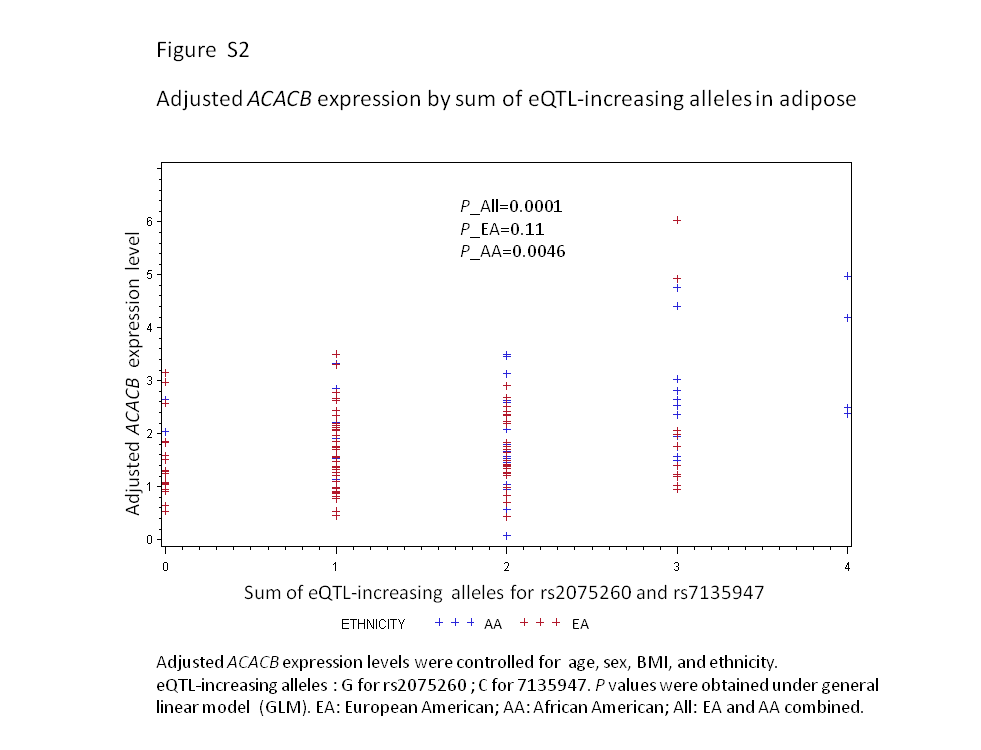

Supplement: Figure S2 — Adjusted ACACB expression by sum of eQTL-increasing alleles in adipose. Adjusted ACACB expression levels were controlled for age, sex, BMI, and ethnicity. eQTL-increasing alleles: G for rs2075260; C for 7135947. P values were obtained under general linear model (GLM). EA: European American; AA: African American; All: EA and AA combined. (TIF) [file pone.0023860.s002.tif]
